# A Guide to the Design of Occupational Safety and Health Training for Immigrant, Latino/a Dairy Workers

**DOI:** 10.3389/fpubh.2016.00282

**Published:** 2016-12-23

**Authors:** Lauren M. Menger, John Rosecrance, Lorann Stallones, Ivette Noami Roman-Muniz

**Affiliations:** ^1^Department of Psychology, College of Natural Sciences, Colorado State University, Fort Collins, CO, USA; ^2^Department of Environmental and Radiological Health Sciences, College of Veterinary Medicine and Biomedical Sciences, Colorado State University, Fort Collins, CO, USA; ^3^Department of Animal Sciences, College of Agricultural Sciences, Colorado State University, Fort Collins, CO, USA

**Keywords:** dairy industry, immigrant Latino/a workers, occupational safety and health, health education, safety training

## Abstract

Industrialized dairy production in the U.S. relies on an immigrant, primarily Latino/a, workforce to meet greater production demands. Given the high rates of injuries and illnesses on U.S. dairies, there is pressing need to develop culturally appropriate training to promote safe practices among immigrant, Latino/a dairy workers. To date, there have been few published research articles or guidelines specific to developing effective occupational safety and health (OSH) training for immigrant, Latino/a workers in the dairy industry. Literature relevant to safety training for immigrant workers in agriculture and other high-risk industries (e.g., construction) was examined to identify promising approaches. The aim of this paper is to provide a practical guide for researchers and practitioners involved in the design and implementation of effective OSH training programs for immigrant, Latino/a workers in the dairy industry. The search was restricted to peer-reviewed academic journals and guidelines published between 1980 and 2015 by universities or extension programs, written in English, and related to health and safety training among immigrant, Latino/a workers within agriculture and other high-risk industries. Relevant recommendations regarding effective training transfer were also included from literature in the field of industrial–organizational psychology. A total of 97 articles were identified, of which 65 met the inclusion criteria and made a unique and significant contribution. The review revealed a number of promising strategies for how to effectively tailor health and safety training for immigrant, Latino/a workers in the dairy industry grouped under five main themes: (1) understanding and involving workers; (2) training content and materials; (3) training methods; (4) maximizing worker engagement; and (5) program evaluation. The identification of best practices in the design and implementation of training programs for immigrant, Latino/a workers within agriculture and other high-risk industries can inform the development of more effective and sustainable health and safety training for immigrant, Latino/a dairy workers in the U.S. and other countries.

## Introduction

Compared to other industries, workers in the U.S. dairy industry experience higher rates of work-related illnesses and injuries (1–3). Aspects of dairy work that pose a threat to worker health and safety include operating hazardous machinery, performing dangerous livestock handling tasks, exposure to hazardous substances (e.g., manure, dirty water, dust, various chemicals), environments prone to slips, trips, and falls, and tasks requiring high repetition, awkward postures, and inadequate rest (1, 4). The U.S. dairy industry relies on an immigrant, primarily Latino/a workforce to meet the demands of operating large-herd, industrialized farms (5, 6). Although detailed surveillance data documenting the patterns of occupational injuries, illnesses, and fatalities in the dairy industry are lacking (7), high rates of injuries and illnesses among immigrant workers have been reported (8–10). Given the direct and indirect expenditures associated with workplace injuries and illnesses, including the high costs of workers’ compensation, and the social burden of injuries and illnesses on individual workers, their families, and communities, it is essential to ensure provision of optimally designed occupational safety and health (OSH) training for immigrant, Latino/a workers in the dairy industry (9).

There are few federal regulations surrounding OSH training in the dairy industry. The Agricultural Exceptionalism law, which was put in place as a way to protect family farms but continues to shield large-scale industrial agriculture, deems the dairy industry exempt from many of the standards enforced in other industries (11). Due to this lack of regulation, OSH training on U.S. dairies is scant, varied in terms of content and scope, and not applied universally (12–16). Román-Muñiz et al. (13) interviewed 72 workers representing 15 dairies to better understand current training available on Colorado dairies. Nearly three quarters reported receiving task-related training (73.6%), while just over half (56.9%) reported receiving safety training upon being hired. Of great concern, nearly one-fifth (19.4%) claimed to have received no job or safety training at all. Focus group studies with dairy workers in Wisconsin (14) as well as in Colorado and South Dakota (15) also found insufficient OSH training. Sorge et al. (16) surveyed dairy producers to assess current cattle handling training on Minnesota dairy farms, of which approximately 25% reported providing no training to new employees. The most commonly mentioned barriers to training were time limitations (39.4%) and language barriers (26%).

It seems that even when OSH training is provided on dairies, it may not have the intended impact on health and safety outcomes. For instance, the previously mentioned study by Román-Muñiz et al. (13) did not find a significant relationship between safety training and injury outcomes. This is not surprising given that training on U.S. dairy farms is often not adequately tailored for an immigrant, Latino/a workforce (10, 12). The majority of immigrant, Latino/a dairy workers have limited formal education, speak little or no English, and have low levels of literacy (17). Many also have limited or no previous dairy experience or foundational training on OSH and, as a result, fail to recognize the hazards in their work (7, 18–20). Immigrant workers also experience a number of unique psychosocial stressors, such as social isolation (21, 22), poverty (22–25), discrimination (15, 22), and lack of employment security (26), that need to be understood and taken into account when developing OSH programs. Such workers may be more likely to take risks at work and less likely to use safety equipment, follow safety procedures, and voice concerns about unsafe conditions (10).

There are few published research articles and guidelines specific to developing effective OSH training programs for immigrant, Latino/a workers in the dairy industry (27). To address this gap, literature relevant to safety training for immigrant, Latino/a workers in agriculture and other high-risk industries was reviewed to identify current practices, promising approaches, and recommendations. The aim of this review is to provide a practical guide for researchers and practitioners involved in the design and implementation of OSH training programs for immigrant, Latino/a workers in the dairy industry. Future interventions will likely be more successful if they build on lessons from the past (28).

## Materials and Methods

The literature search was restricted to peer-reviewed academic journal articles and guidelines that were (1) published between 1980 and 2015; (2) by universities or extension programs; (3) written in English; and (4) related to OSH training among foreign-born immigrant, Latino/a workers within agriculture and other high-risk industries. The following databases were used for the search: Academic Search Premier, Agricola, CAB Abstracts, ERIC, MEDLINE, PsycInfo, and Web of Science. Three categories of search terms were used as follows: (1) OSH training (i.e., training, safety training, safety education, industrial safety); (2) immigrant, Latino/a workers (i.e., immigrant, migrant, Hispanic, Latino/a, Spanish-speaking worker, foreign born); and (3) agriculture and other high-risk industries (i.e., dairy, farming, agriculture, construction, forestry, mining, transportation, high-risk industry). A Boolean search of article abstracts included terms from each category in an additive manner (AND) while including terms within each category in a disjunctive manner (OR).

The above criteria were used to select only abstracts showing a relationship to the topic. First, article abstracts were reviewed to assess applicability. If abstracts included insufficient information to determine relevance, then the full text was reviewed. Reference lists from published papers were also screened to identify additional articles not identified in the initial search. Articles were excluded from further analysis if they (a) did not concern immigrant, Latino/a workers and (b) did not provide suggestions or insights related to OSH training. A summary of recommendations to promote training transfer, defined as the transfer of the knowledge and skills learned in training to the work context, were also included from literature in the field of industrial–organizational psychology.

## Results

The review uncovered a number of articles related to designing OSH training for immigrant, Latino/a dairy workers. A total of 97 articles were identified, of which 65 are cited in Sections “Results” and/or “Discussion”. Thirty-two of the identified articles were not included either because they did not meet the inclusion criteria upon review or because they did not make a unique and significant contribution beyond those already cited. Articles cited include literature reviews, intervention studies, survey studies, qualitative studies, scale validation studies, and case studies, as well as publications regarding training transfer best practices. Additional articles are cited to provide relevant context and background information. The literature on OSH recommendations was categorized by five main themes: understanding and involving workers, training content and materials, training methods, maximizing worker engagement, and program evaluation. Following is a description of each theme with relevant recommended strategies. See Table 1 for a summary of recommended strategies.

**Table 1:** **Summary of recommended approaches and example of key strategies for each theme**.

Theme	Recommended approaches	Example key strategies (citation/s)
Understand and involve workers	Formative research	Include dairy management and workers in the process (7, 28, 33)
	Community-based participatory methods	Consider the goals/strengths of all stakeholders (35) Provide multiple avenues for participation (35)
Training content and materials	Be comprehensive	Train all workers on OSH across all areas of the dairy (7, 41) Focus on the how *and* why of safety procedures (42, 43) Provide resources for where to get help with OSH (46) and non-work (22) issues
Be language and literacy appropriate	Provide materials in workers’ native language/s and keep materials at a low reading level (56) Use realistic and common symbols, objects, and settings (51, 52) Use various strategies [e.g., a decentering translation approach (49), field testing (58), cognitive interviewing (53), collaboration with ESL and literacy professionals (54), provision of learning aids (55, 56)] to ensure comprehension
Embrace cultural diversity	Understand and incorporate workers’ core cultural beliefs (49, 60), avoid stereotypes, and remain sensitive to varying levels of acculturation (49) Include familiar culture phenomenon, same race/ethnicity role models, and deliver materials in a way workers are accustomed to receiving information (29, 58)
Acknowledge workers’ realities	Ensure materials reflect day-to-day realities of the workplace (15) and the rapid pace of change in the dairy industry (27)
Training methods	Use a variety of formats and media	Use multiple formats/media to accommodate low literacy and different learning preferences (7, 65)
	Promote active participation	Reinforce training content through quizzes and games rather than written formats (44) Tailor methods to cultural attitudes toward learning and other cultural factors (44, 74)
	Empower workers	Allow workers to develop their own OSH goals and action plans (28) Foster leadership skills for organizing and taking action (44)
	Enlist peers as trainers	Adopt the *Promotoras de Salud* model (79–82)
	Promote training transfer	Foster motivation (43, 85, 93) and efficacy to apply training content (84, 86) Highlight situational cues within and outside of the workplace (78) Provide additional learning and practice opportunities after training (84)
Maximize engagement		Consult workers regarding training logistics (75, 89) Encourage dairy management to foster a strong culture of OSH through their words and actions (7) Treat OSH training as an ongoing process rather than a one-time event (73)
Program evaluation		Evaluate the impact of the program as well as the process Utilize quantitative, qualitative (99, 100), and longitudinal methods when possible

### Understand and Involve Workers

Among immigrant, Latino/a workers, there is great variation in terms of country of origin, level of education and acculturation, language and literacy skills, current and past socioeconomic status, legal status, and experiences of racism and discrimination (29). Even workers who come from the same country can have vastly different life experiences and occupational backgrounds (7) and varying exposure to OSH hazards and safety training (30). In order to develop effective and appropriately tailored OSH training, it is essential to understand the diverse realities of immigrant, Latino/a workers. Formative research and community-based participatory (CBP) methods are two promising approaches for understanding workers through active involvement.

#### Formative Research

Formative research aims to understand the knowledge attitudes, opinions, skills, beliefs, and needs of the target population in relation to the health issues of interest and can take many forms, including literature reviews, health assessments, observations, interviews, and focus group discussions (31, 32). Formative research should include both dairy management and the workers themselves to identify key issues, ensure relevance, and gain buy-in through both bottom–up and top–down approaches (7, 33). If OSH programing is developed based on the expressed needs and concerns of dairy management and the workers themselves, they will feel their voice has been heard and may be more inclined to actively engage in it (or in the case of dairy management, promote engagement in it) as a result (34).

#### CBP Methods

CBP methods have been suggested as a way to continue to engage managers and workers as key decision makers throughout the entire OSH program design, implementation, and dissemination process (35). The aim of taking a CBP approach is to bring together members from the target group and program developers in order to “establish trust, share power, foster co-learning, enhance strengths and resources, build capacity, and examine and address community-identified needs and health problems” (36) (p. 14). There are many ways to adopt a participatory approach. For instance, workers can assist in the development of training materials and company documents and policies related to OSH, the latter of which often include legal and technical jargon that would confuse even the most educated and experienced readers (37). CBP methods facilitate increased access to and trust with community members and result in programs that are more culturally and educationally appropriate, sustainable, and replicable in other communities (35). CBP approaches capitalize on the valuable insights and collective strengths of the target group while also developing their capacity to advocate for and achieve change (35). Arcury et al. (35) reviewed five CBP programs aiming to prevent pesticide exposure among migrant and seasonal farmworkers to identify common elements of successful CBP projects. They emphasized the importance of taking the time to build and maintain relationships with and understand the goals and strengths of all stakeholder groups concerned with promoting OSH (e.g., workers, family members, farmers, general community residents, advocacy groups, healthcare providers), being flexible and creative, and providing multiple avenues for participation so different stakeholders can get involved to their own ability/comfort levels. See Quandt et al. (38) for an example of a CBP approach to develop a pesticide safety program with Mexican farmworkers in North Carolina.

### Training Content and Materials

In addition to being based on sound health communication principles [e.g., see Rowan (39) for risk communication guidelines], OSH training content and materials must be tailored to the unique needs of immigrant, Latino/a dairy workers. Overall, content and materials should be comprehensive and appropriately tailored in terms of language and literacy, cultural diversity, and workplace realities.

#### Be Comprehensive

Many immigrant workers have limited or no previous dairy experience (14, 15, 40), and therefore OSH programs should be comprehensive in terms of covering all potential hazards (7). Overall, it should not be assumed that workers have even basic OSH knowledge. In addition to safe animal handling, operation of hazardous machinery, and prevention of environmental risks (e.g., heat-related illnesses), dairy workers should also be trained on body mechanics and ergonomics to prevent musculoskeletal disorders common in industrialized dairy (41). OSH training should cover both the how and why of protective measures and all workers should be trained equally, regardless of previous experience, as task and safety procedures vary across dairies (42, 43). Workers should be trained in safety for all areas of the dairy, especially when there is a tendency for job rotation. Trainings should also educate workers about health insurance and workers compensation (14, 15), as well as their rights and responsibilities under OSHA, and provide information and resources on where to get help in addressing OSH problems (44, 45). Additionally, trainings should include information pertaining to employer responsibilities and how workers can go about pursuing grievances when these responsibilities are not fulfilled (46).

Finally, OSH programs should also be holistic in the sense of acknowledging non-work-related challenges faced by immigrant, Latino/a workers. For instance, financial strain, separation from family, and poor work–life balance could lead to stress and family dysfunction, which in turn could affect productivity and safety at work (21). Workers should be provided with information regarding affordable and accessible community resources and services available to help cope with some of these non-work issues (22).

#### Be Language and Literacy Appropriate

With few exceptions [e.g., Ref. (33, 47)], language and literacy appropriate OSH trainings for immigrant, Latino/a workers are rare. This poses a serious problem: if workers cannot understand the content and materials, they will be minimally effective in promoting OSH. In terms of language, training materials should be provided in Spanish and English (and/or another language native to workers) to accommodate workers who may feel more comfortable with one language than the other (48). When translating materials, an approach known as *decentering*, which prioritizes conceptual clarity over literal translation, has been recommended [see Brunette (49) for a more detailed description]. Care should be taken to use tildes and accents as appropriate, as they influence the meaning of many Spanish words (48). Immigrant, Latino/a farmworkers have been found to have an average of 6 years of education; thus, it is generally recommended to keep materials at a low reading level (50). Materials including realistic and vivid symbols with well-known objects and settings and the use of traffic light colors to communicate risk level have been suggested for use with Latino/a farmworkers (51, 52).

To ensure that materials are at the appropriate level, they should be field tested with members of the target audience who are not able to fluently read or write in either language (44). Cognitive interviewing, in which members of the target group are given the training materials and asked to articulate their understanding of the information in order to determine if the intended messages are being communicated, may be a particularly useful approach in this context (53). Health and safety educators can also collaborate with ESL and literacy professionals to ensure that content is at the right level (54). To ensure comprehension of common dairy industry terms, McGlothlin et al. (55) recommended providing workers with a pictorial glossary with a description of basic and advanced terminology to refer to during training as well as after training is complete. Similarly, Opatik and Novak (56) recommended provision of a pocket size pronunciation guide.

#### Embrace Cultural Diversity

Simple translation of OSH materials with subtitles in the necessary language(s) and at the appropriate level is not sufficient (49, 57). On a basic level, training content should incorporate familiar cultural phenomenon. For instance, materials should include same race/ethnicity role models and be packaged in a way that is similar to how workers are accustomed to receiving information in their home countries (58). At the same time, it is important to avoid cultural stereotypes and remain sensitive to varying levels of acculturation. Members from the target population should review materials to confirm cultural relevance (49).

Beyond aligning training with familiar cultural phenomenon and imagery, concerted effort should be made to understand and incorporate the core cultural beliefs of the workers (especially those that may affect OSH) into training materials (49). Latinos/as tend to share a common set of values that are different from those found in mainstream American culture, such as higher levels of in-group collectivism, greater acceptance of hierarchical power structures, and stronger adherence to traditional gender roles (59, 60). For instance, *familism* (i.e., a strong attachment to nuclear and extended family) is a central value among Latinos/as that may influence OSH behaviors (60). Training content and materials should emphasize the family-related implications of not adhering to OSH policies and procedures. Immigrant workers also have diverse cultural beliefs related to health behaviors, which need to be understood and integrated into OSH training (61, 62). Training should sensitively emphasize the implications of adhering to potentially risky traditional practices and beliefs (63). See Sanders-Smith (48) for a review of cultural issues that impact the Latino/a workforce.

#### Acknowledge Workplace Realities

It is essential that OSH training is reflective of workers’ day-to-day realities (15). When safety information is viewed as meaningful, valuable, and relevant to everyday experiences, it may enhance motivation to learn and aid with memorization and recollection processes (63). One way to ensure OSH training reflects workplace realities is to integrate it with task training as much as possible (13). Whether delivered separately or in conjunction with task training, careful attention should be made to acknowledge what is feasible or realistic for the workers. For instance, merely informing workers of safety policies and procedures does not acknowledge the fear of negative consequences workers may have if they act on or report hazardous situations (7). It is also important to regularly update and adapt OSH trainings to coincide with the rapid pace of change in organizational practices in the dairy industry, such as changes in globalization, migration patterns, and the economy (27).

### Training Methods

In addition to training content, the methods by which OSH training programs are delivered must also be carefully tailored to meet the unique needs of immigrant, Latino/a dairy workers. Overall, native Spanish-speaking trainers are suggested over English-speaking trainers with translators given the difficulty in ensuring accurate translation (64). Ideally, trainers should be bilingual *and* bicultural so that they can aid in promoting comprehension of OSH concepts while adjusting for relevant cultural influences (58). A few basic principles to follow are to use a variety of formats and media, promote active participation, empower workers, enlist peers as trainers, and promote training transfer.

#### Use a Variety of Formats and Media

It is likely that some workers may not have developed the skills to learn through formal education and, therefore, may have limited ability to learn new concepts through traditional pedagogical approaches. A variety of formats (e.g., audio-visual, face to face, verbal communication, hands-on) and media (e.g., flipcharts, videos, comic books, cartoons, *fotonovelas*, targeted brochures) should be utilized to accommodate low literacy levels and different learning preferences (7). See Reinhardt et al. (65) for an example of a training using various formats and media in the context of dairy OSH. One creative approach that has proven to effectively increase health-related knowledge among immigrant, Latino/a farmworker populations is the use of theatrical presentations to disseminate health information [e.g., Ref. (66, 67)]. For instance, Holmes et al. (41) found a combined a Spanish language *fotonovela* play, a live demonstration and practice session, and educational pamphlets effective in promoting correct lifting techniques among predominantly female, Latino/a fruit warehouse workers.

Computer-based instruction may be considered as a more cost efficient training modality compared to in-person training. Anger et al. (68, 69) found support for using computer-based instruction in promoting OSH knowledge and behavioral outcomes among Latino/a workers with limited formal education. Evia (70) proposed a participatory approach to design culturally tailored computer-based OSH training for Latino/a construction workers. Mobile phone and internet-based interventions may also be promising modes of OSH training, but may be more effective with younger working populations (71).

#### Promote Active Participation

There is a large literature base suggesting that trainings based on active participation are more effective than lecture-based trainings (72). There are numerous ways to promote active engagement in trainings, such as group problem solving, hazard mapping, hands-on demonstrations and simulations, role-playing activities, photo voice, and other art-based approaches (see O’Connor et al. (44) for a more detailed list of approaches and Román-Muñiz et al. (73) for examples of hands-on demonstrations in the dairy context). In reviewing training content, verbal quizzes and games should be used instead of written formats to reinforce training messages and to invite discussion or questions (44). Trainees should also be provided with regular feedback so that they can evaluate their progress and learn from their mistakes (44).

It is also important to consider cultural attitudes toward learning and adjust training methods accordingly. For instance, Latino/a workers may perceive expressing dissenting opinions or asking questions as disrespectful, so instructors may need to make extra efforts to encourage active participation (74). For example, it may be necessary to break trainees into small groups to make them feel comfortable discussing issues and sharing their experiences (44). Cultural variation in gender dynamics should also be considered. For instance, trainees may feel more comfortable sharing if grouped by gender (44). Given the strong value placed on family within Latino/a culture, it may also be beneficial to determine ways to involve family members in the learning process. For instance, workers can be sent home with information to share or an activity to work on with their family.

#### Empower Workers

OSH training will be minimally effective if workers do not feel empowered to implement the lessons learned. There are a number of ways in which trainings can be tailored to empower workers to stay healthy and safe. At the individual level, OSH training activities should empower workers to identify and analyze OSH issues and develop their own solutions and action plans (44). Hurley and Lebbon (9) suggested that workers regularly meet with managers to set safety-related goals, which would also serve to further strengthen worker–manager relations. Assertiveness training may also be beneficial in terms of helping dairy workers to overcome fears of speaking up regarding OSH issues and concerns (75). However, Shrestha and Menzel (76) piloted an assertiveness training as part of a fall prevention training targeting Latino/a construction workers and found that a low percentage of workers identified the training as useful. They attributed this finding to the possibility that workers may view speaking up about safety issues as a threat to their job security, which is often prioritized over safety. At the collective level, training should present safety as a shared responsibility, encourage workers to discuss risk and protective factors with one another, and promote leadership skills for organizing and taking action in terms of advocating for provision of personal protective equipment and remediation of hazards in the work environment (44, 77, 78).

#### Enlist Peers as Trainers

Immigrant, Latino/a dairy workers may be more likely to learn about OSH from their coworkers than from formal training programs. Román-Muñiz et al. (13) found that training provided by coworkers had a protective effect against work-related injuries, but this effect was not found when provided by dairy managers. They speculated that coworkers may be better able to deliver safety information in an informal and culturally acceptable manner, compared to managers who may have inadequate Spanish language skills and/or a limited comprehension of the cultural factors that influence effective communication (p. 23).

The *Promotoras de Salud* model is an approach that relies on trained lay Latino/a community members to deliver health messages and has been found to be effective in changing OSH attitudes and behaviors of immigrant, Latino/a workers in high-risk jobs [e.g., Ref. (79–82)]. Bush et al. (81) piloted a *promotoras* program with forest workers and found increased knowledge and awareness of OSH risks and resources. They highlighted the importance of providing non-literacy-based outreach and training tools and leadership development opportunities for *promotoras* and engaging *promotoras* in community outreach to connect with workers in comfortable environments (e.g., homes, community festivals, churches). It is also important that *promotoras* be frequently praised; educated regarding local, state, and national events affecting immigrant workers; supported in troubleshooting personal, community, and work-related obstacles; and provided with an honorarium to make the investment of their time worthwhile and foster a sense of accomplishment (82). The value of adopting participatory approaches, both in terms of training methods and project planning has been emphasized in order to promote effectiveness, commitment, and leadership skills among peer trainers (83).

Despite the many benefits of peer trainers, some workers may prefer to receive training from an expert because: (1) an expert might be viewed as more knowledgeable and therefore be taken more seriously and (2) a trained peer might leave causing a sudden loss of benefit from their knowledge (38). Therefore, it could be beneficial to use a combination of peer and expert trainers.

#### Promote Training Transfer

Training transfer, a construct from the field of industrial–organizational psychology, is the extent to which the knowledge and skills learned in training are transferred to the job (84). In order to promote the transfer of training, first trainees must feel motivated to learn and have efficacy to apply training content to the workplace (84). Latino/a workers’ perceptions of work as essential to life and pain as an inevitable part of work (61) and beliefs that animal-related injuries are not preventable (15) may cause them to be less motivated to engage in the preventive behaviors promoted in OSH programing. Special efforts may be needed to supersede these beliefs and help workers understand the efficacy and value of prevention. The goal should be to help workers develop motivation, confidence, and critical thinking skills to apply OSH training content to protect themselves from the myriad hazards they face in their work. Some programs offer incentives, such as a completion certificates, to motivate training transfer [e.g., Ref. (43, 75)]. As previously suggested, internal motivation can be fostered by personalizing OSH training content to the day-to-day realities faced by workers and managers to ensure that they perceive it as relevant and useful (85). Self-efficacy can be promoted through behavioral modeling (including both positive and negative examples of desired behaviors) and providing opportunities for trainees to practice using new knowledge/skills (86). Role-playing exercises and other types of simulations can be beneficial in terms of promoting active learning and providing opportunities to practice (84). It is also important to help trainees recognize the challenges they may face in implementing the new knowledge and skills and brainstorm strategies to overcome them through error management techniques (87).

Training should be conducted in an environment that closely resembles the workplace to make for a more natural transition from the training context to the work context (84). Kraiger et al. (84, 88) recommended varying training scenarios to help trainees develop the skills they will need to handle issues across multiple conditions that can occur on the job. Training should also highlight situational cues within and outside of the workplace that will help trainees remember to engage in OSH behaviors (78). For instance, Quandt et al. (78) encouraged trainees to lay a mat outside the door of their home as a visual reminder that work boots should be removed before entering to keep their children and other family members safe from harmful exposures. To promote sustained training transfer, it is also important for trainings to foster supervisor and peer support to engage in OSH behaviors and provide additional learning opportunities after training (e.g., through refresher training and after action reviews) (84).

### Maximize Worker Engagement in OSH Programing

If workers are not engaged in OSH initiatives, they are not likely to benefit from them. In order to maximize engagement, it is important to acknowledge that workers may not view safety as their number one priority and build OSH content into trainings on other topics that workers view as higher priorities (which can be identified through formative research) (44). Workers should be consulted regarding the ideal duration and scheduling of OSH trainings (75, 89). A high level of engagement can also be promoted by collaborating with community organizations that already have a well-established relationship with the immigrant, Latino/a community [e.g., Ref. (33, 90)]. To ensure continued engagement, it is also important that OSH training be treated as an ongoing process that all workers, regardless of tenure, are encouraged to participate in, rather than a one-time event (73).

Another key approach to enhancing worker engagement is for dairy owners/managers to foster a strong culture of OSH throughout the dairy. Due to the frequency of their exposure to OSH hazards, dairy owners/managers may deny susceptibility to risk or be skeptical of safety measures and give workers the impression that the health and safety of the cows is prioritized over that of the workers (7). Programs are needed to educate dairy owners/managers about the realities concerning various risks inherent in dairy work, the impact of poor OSH on productivity and the bottom line (7), and how to foster an atmosphere of trust in which workers feel their safety is a priority. Dairy owners and managers should be actively involved in trainings, which will in turn enhance perceived safety culture and inspire workers to transfer the OSH knowledge and skills learned to the workplace (7, 73). It may be beneficial to start by assessing safety climate, a measure of safety culture, to identify areas of strength and opportunities for improvement. Measuring changes in safety climate across time can also be used to assess the impact of OSH interventions [see Jorgensen et al. (91) and Flynn (92) for more on assessing safety climate among workers from different backgrounds].

### Program Evaluation

OSH programs in the agricultural sector have historically lacked rigorous program evaluation (12, 93). This deficiency greatly limits the potential of such programs to contribute to the evidence base of what does and does not work in terms of protecting and promoting worker health. To ensure the optimization of OSH training for immigrant, Latino/a dairy workers, program evaluation is critical. In addition to providing evidence of programmatic worth, evaluation can be used to identify strengths and weaknesses, examine the extent to which goals are being met, and supply information that can be used to improve program outcomes (94–97). Evaluations should assess whether OSH programs are having the intended impact on worker attitudes and behaviors as well as incidents of work-related injuries and illnesses (i.e., impact evaluation) and collect the views of dairy workers and management related to program strengths and weaknesses and suggestions for improvement (i.e., process evaluation). Some have suggested conducting process evaluation during the implementation phase so feedback can be used to make improvements before the end of the program [e.g., Ref. (98)]. It may be necessary to provide bilingual staff members to assist non-literate participants in completing evaluation materials verbally (41, 75).

Whether conducting an impact or process evaluation, both quantitative and qualitative approaches (i.e., mixed methods) should be utilized to provide a richer view of the knowledge level, attitudes, and practices of low literacy workers (99). Ahonen et al. (100) used a participatory, mixed methods approach to evaluate the design, delivery, reactions, participant learning, application of skills, dissemination, strengths and weakness, and return on investment of an OSH training with Spanish-speaking immigrant construction workers. Longitudinal methods are beneficial for assessing longer term program impacts but may be challenging due to high rates of turnover in the dairy industry. See Vela-Acosta et al. (33) for another example of a mixed methods evaluation approach used with Latino/a farmworkers and DeRoo and Rautiainien (12) and O’Connor et al. (44) for further recommendations related to program evaluation.

## Discussion

Despite increasing reliance on immigrant, Latino/a workers within the dairy industry, few studies to date have focused on OSH training for this high-risk group. In order to fill this gap, the goal of this review was to summarize current strategies embraced by OSH training for immigrant, Latino/a workers from agriculture and other high-risk industries in order to provide a practical guide for researchers and practitioners involved in the design and implementation of effective OSH training programs for immigrant, Latino/a workers in the dairy industry. Overall, whether programs are developed from the ground up or borrowed from elsewhere, it is essential that training materials and methods be based on a deep understanding the characteristics and realities of the immigrant worker (58). Strategies must also be adopted to maximize worker engagement and, in turn, program impact on OSH outcomes.

Of concern, this review revealed that many OSH programs targeting immigrant, Latino/a workers in high-risk industries fail to embrace established cultural models and systematic program and process evaluation. These deficiencies could result in the implementation or replication of programs that are based more on practitioners’ perceptions and intuitions about how to tailor interventions for Latino/a workers than on empirically tested theories. Also of concern is the fact that many programs are one-time events; yet, high rates of turnover and mobility among immigrant dairy workers demand an ongoing approach to training (7).

*Seguridad en las Lecherías* (17) is a recently developed program to promote OSH among immigrant, Latino/a dairy workers in Wisconsin that adopts many of the suggested guidelines. *Seguridad en las Lecherías* is a theory-based program utilizing the *promotoras* model for the first time within the dairy context. The program was developed based on formative research conducted to understand the perspectives and needs of dairy workers and producers. It was designed to be engaging, appropriate for workers with limited formal education and low literacy, and easy to replicate. It was conducted in partnership with various stakeholders (e.g., the Professional Dairy Producers of WI, the Mexican Consulate of St. Paul) who have extant knowledge of and experience with immigrant, Latino/a dairy workers. See Tovar-Aguilar et al. (101) for an example of another program that utilized many of the best practices outlined in this review to promote eye safety among Latino/a citrus harvest workers.

### Limitations and Future Research

There are many important directions for future research related to OSH programing for immigrant, Latino/a dairy workers. First, there is a great need for additional studies to better understand the prevalence and nature of occupational injuries and illnesses among this working population, which can guide the prioritization of OSH programs. In order to acquire accurate epidemiologic data, efforts are needed to improve reporting of work-related injuries, illnesses, and near misses (7). Although the present review identified a number of promising strategies, systematic meta-analyses are also needed to assess the relative effectiveness of intervention approaches that incorporate the specific training needs of the Latino/a workforce.

It is important to keep in mind that training programs are just one component of OSH for immigrant, Latino/a dairy workers. Training programs are limited in that they only focus on the individual worker, rather than addressing the root cause of injuries and exposures (102). Hagevoort et al. (103) have advocated for a macro approach to OSH programing, inclusive of the workers, the work environment, as well as cultural, social, and economic factors external to the work context. Higher level policy changes and their proper enforcement are needed to standardize OSH training for the dairy industry and address the challenging life circumstances of immigrant, Latino/a workers (104, 105). It is also important to promote access to and utilization of health services (106) and ameliorate physical and psychosocial stressors among immigrant, Latino/a workers, which have been associated with decreased cognitive function and mental health-related outcomes (107). OSH initiatives focused on engineering and administrative controls in addition to those focused on individual behavior change are also essential in reducing workplace hazards (108).

Another major difficulty related to promoting OSH among immigrant, Latino/a dairy workers lies in the considerable communication barriers between workers and non-Latino/a dairy owners and managers (89). In addition to OSH training, ESL and SSL classes should be provided to improve communication and comprehension of OSH materials (109). Programs targeting dairy management are also needed to promote safety leadership, engagement in OSH programing, cultural awareness, and skills in building positive and trusting relationships with immigrant, Latino/a workers (110). See Viveros-Guzmán and Gertler (111) for additional suggestions regarding improving communications between immigrant farmworkers and their employers.

## Conclusion

The U.S. dairy industry and its workforce have undergone dramatic transformations in recent decades (14). OSH training programs in the dairy industry must take into account changing workforce demographics and the realities of a global immigrant workforce. Training programs that adapt to the needs of their specific workforce will have the greatest impact and effectiveness. Given the high rates of occupational injuries and illnesses in the dairy industry and the increasing reliance on an immigrant, primarily Latino/a workforce, efforts to protect and promote health and safety must be sensitive to the unique attitudes, understandings, and behaviors of immigrant, Latino/a workers. As stated by Liebman et al. (17), “it is incumbent upon the industry to address the risks associated with bringing a naïve workforce into one of the most dangerous areas (large-animal agriculture) of one of the most dangerous industries (agriculture) in the country” (p. 81). This review marks an initial step in identifying current practices and promising approaches in the design and implementation of OSH training programs for immigrant, Latino/a workers within agriculture and other high-risk industries to inform the development of more effective and sustainable OSH training for immigrant, Latino/a workers in the dairy industry. It is our hope that the programs reviewed provide a significant foundation which researchers and practitioners can challenge, test, and build upon.

## Author Contributions

All authors (LM, JR, LS, and IR-M) have (1) contributed substantially to the conception or design of the work and/or the acquisition, analysis, or interpretation of the data for the work, (2) participated in drafting the work or revising it critically for important intellectual content, (3) approved the final version to be published, and (4) agreed to be accountable for all aspects of the work in ensuring that questions related to the accuracy or integrity of any part of the work are appropriately investigated and resolved. LM and IR-M contributed to the design of the work, analysis and interpretation of the data, and the drafting and revising of the manuscript. LS and JR contributed to the design of the work and the drafting and revising of the manuscript.

## Conflict of Interest Statement

The research was conducted in the absence of any commercial or financial relationships that could be construed as a potential conflict of interest.
